# Gastro-Esophageal Reflux Disease and Paroxysmal Atrial Fibrillation Ablation

**DOI:** 10.3390/life13051107

**Published:** 2023-04-28

**Authors:** Mariana Floria, Diana-Elena Iov, Daniela Maria Tanase, Oana Bogdana Barboi, Genoveva Livia Baroi, Alexandru Burlacu, Mihaela Grecu, Radu Andy Sascau, Cristian Statescu, Catalina Mihai, Vasile Liviu Drug

**Affiliations:** 1 Department of Internal Medicine, Grigore T. Popa University of Medicine and Pharmacy, 700115 Iași, Romania; 2 Internal Medicine Clinic, Saint Spiridon Emergency Hospital, 700115 Iași, Romania; 3 Institute of Gastroenterology and Hepatology, 700115 Iasi, Romania; 4 Department of Vascular Surgery, Saint Spiridon Emergency Hospital, 700115 Iași, Romania; 5 Cardiology Department, Cardiovascular Disease Institute, 700503 Iași, Romania

**Keywords:** atrial fibrillation, sinus rhythm, gastroesophageal reflux disease, ablation, esophagitis

## Abstract

Patients undergoing ablation for atrial fibrillation may be at increased risk of developing gastroesophageal reflux disease. We prospectively studied the presence of symptomatic gastroesophageal reflux disease in naïve patients who underwent atrial fibrillation ablation. Methods: The presence of typical symptoms suggestive of gastroesophageal reflux disease was clinically assessed by the gastroenterologist at baseline and at 3 months after ablation. In addition to that, all patients underwent upper gastrointestinal endoscopy. Results: Seventy-five patients were included in two groups: 46 patients who underwent atrial fibrillation ablation (study group) and 29 patients without ablation (control group). Patients with atrial fibrillation ablation were younger (57.76 ± 7.66 years versus 67.81 ± 8.52 years; *p* = 0.001), predominantly male (62.2% versus 33.3%; *p* = 0.030) and with higher body mass index (28.96 ± 3.12 kg/m^2^ versus 26.81 ± 5.19 kg/m^2^; *p* = 0.046). At three months after the ablation, in the study and control groups, there were 88.9% and 57.1% patients in sinus rhythm, respectively, (*p* = 0.009). Symptomatic gastroesophageal reflux disease was not more frequent in the study group (42.2% versus 61.9%; *p* = 0.220). There was no difference in terms of sinus rhythm prevalence in patients with versus without symptomatic gastroesophageal reflux disease (89.5% versus 88.5%; *p* = 0.709). Conclusion: In this small prospective study, typical symptoms suggestive of gastroesophageal reflux disease were not more frequent three months following atrial fibrillation ablation.

## 1. Introduction

Gastroesophageal reflux disease and atrial fibrillation currently represent two of the most important health issues across the globe. Atrial fibrillation is the most common cardiac arrhythmia encountered in adults worldwide, with significant associated morbidity and mortality [1]. Pulmonary vein isolation by catheter ablation has been proposed as a curative method, with current American and European Guidelines endorsing this technique as a method of rhythm control in patients with symptomatic paroxysmal atrial fibrillation [2,3]. Studies investigating the effectiveness of this method suggest that in patients fit for the procedure, it should be regarded as the first-line treatment, given its potential to significantly reduce atrial tachyarrhythmia recurrence compared to antiarrhythmic medication [4]. This technique is used more and more frequently in young symptomatic patients, becoming the standard in paroxysmal atrial fibrillation therapy all over the world.

Although catheter ablation is regarded as a minimally invasive procedure, it is not entirely without risks [5,6,7,8,9]. Given the anatomical proximity between the posterior wall of the left atria and the esophagus, thermal esophageal lesions are possible early complications following atrial fibrillation ablation [9]. These may range in severity from erythema and esophagitis to ulceration, necrosis and, even, atrio-esophageal fistula formation [5,6,7,8,9]. These esophageal ulcerations in the proximity of the posterior left atrial wall may be influenced by the radiofrequency lesion set and maximum energy delivered. Temperature monitoring, active cooling or visualization of the esophageal anatomical position have been studied as possible solutions to protect the esophagus during the radiofrequency catheter ablation procedure.

In addition to that, studies also reported sporadic new onset gastroesophageal reflux following this interventional ablation technique targeting atrial fibrillation [8]. This phenomenon is hypothesized to be due to potential damage to the vagal plexus surrounding the distal esophagus during the ablation procedure [5]. Given this possible association, acid-suppressive medications have been proposed as a potential preventive strategy. However, recent studies have yielded inconsistent results that may challenge this recommendation [10,11]. Therefore, prophylactic treatment with proton pomp inhibitors (for a maximum duration of 4 weeks) for all patients undergoing radiofrequency catheter ablation for atrial fibrillation is currently still a controversial topic. In addition to that, a recent meta-analysis of observational studies set out to investigate the association between gastro-esophageal reflux and atrial fibrillation, but did not reach a conclusive result, suggesting that more research is still much needed in this field of research [12].

The aim of this study was to assess the presence of typical symptoms suggestive of gastro-esophageal reflux disease in naïve patients who underwent catheter ablation for paroxysmal atrial fibrillation, at baseline and at three months following the procedure.

## 2. Materials and Methods

### 2.1. Study Subjects

We aimed to investigate the presence of symptomatic gastro-esophageal reflux at baseline and at 3 months after radiofrequency catheter ablation for atrial fibrillation. This pilot study enrolled patients from the Cardiovascular Diseases Institute for the duration of one year. Subjects were evaluated by a multidisciplinary team consisting of Gastroenterology and Cardiology specialists.

Inclusion criteria were defined as follows: adult patients (>18 years old), with documented non-valvular paroxysmal atrial fibrillation were referred for endocardial catheter ablation (the indication for the procedure was established in accordance with current guideline recommendations [13]: younger, otherwise healthy patients with symptomatic paroxysmal atrial fibrillation refractory to at least one antiarrhythmic medication, taking into consideration the patient preference for the technique), without any prior gastroenterological evaluation or treatment (naïve patients) and who provided signed informed consent. All patients included in the study group underwent transesophageal echocardiographic evaluation and cardiac computerized tomography, as well as the recommended preprocedural work up [14].

Exclusion criteria were defined as follows: patients with known chronic gastrointestinal disorders; patients with acid-suppressive treatment (e.g., proton pump inhibitors); patients with any valvular disease more than mild thyroid disorders, a personal history of myocardial infarction, transient ischemic attack or stroke; patients with an implantable defibrillator or pacemaker; patients with known inflammatory diseases or under immunosuppressive therapy; patients with active neoplasia; patients with dementia or other invalidating psychiatric pathology; patients treated with non-steroidal anti-inflammatory drugs (including acetylsalicylic acid in a dose greater than 100 mg per day) at enrollment and in the prior 30 days; patient refusal.

**Figure 1:**
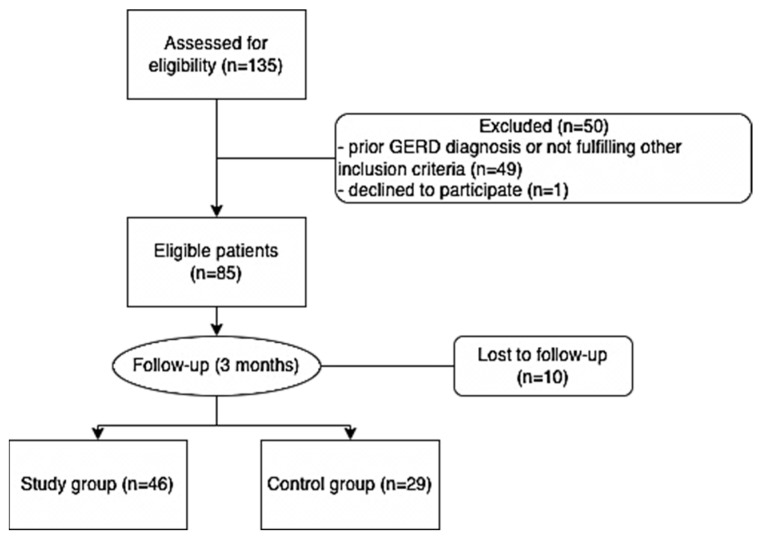
Flowchart of the study participants.

The control group consisted of subjects with paroxysmal atrial fibrillation who received only standard medical treatment. These patients did not undergo atrial fibrillation ablation, either because they were not suitable candidates for the procedure, or due to patient preference. The controls were recruited from the same clinic and throughout the same enrollment period as the patients from the study group. A flowchart of the study participants is presented in Figure 1.

Non-valvular paroxysmal atrial fibrillation was diagnosed in patients without mitral stenosis or artificial heart valves, according to current guidelines [14]. It was defined as ECG or Holter ECG-documented atrial fibrillation that terminated spontaneously or with intervention within 7 days of onset [14].

Symptomatic gastro-esophageal reflux was clinically diagnosed by a gastroenterologist in accordance with the Montreal definition [15]. It was defined as the presence of mild typical symptoms of heartburn and/or regurgitation at least 2 times per week, or moderate/severe symptoms occurring more than once a week, perceived as “troublesome” by patients [15]. 

### 2.2. Study Design

A detailed medical history was obtained for each study participant and the following clinical parameters were noted: age, gender, smoking status, the presence of obesity (defined as a body mass index higher than 30 kg/m^2^), dyslipidemia, hypertension, diabetes mellitus, heart failure, ischemic heart disease and peripheral arterial disease.

Among the usual biological parameters, pro-BNP (brain natriuretic peptide) was assessed in the study participants as a marker of left atrium structural remodeling. 

All included subjects underwent gastroenterological evaluation (by upper gastrointestinal endoscopy) and cardiological assessment (by 24 h Holter ECG monitoring and bidimensional transthoracic echocardiography) 48–72 h before (baseline values) and 3 months after the atrial fibrillation ablation procedure. Heart rhythm was recorded by 24 h ECG Holter monitoring using a two-channel tracker (EC-2H 2-Channel, Cardiospy, LabtechHolter ECG System, Labtech Ltd., Hungary). The cardiologists counselled patients to report symptoms of arrhythmia between scheduled visits (before and three months after the atrial fibrillation ablation procedure).

Following the ablation procedure, no dietary recommendations have been made to the patients. Likewise, no acid-suppressive medications have been prescribed to the study participants in the 3-month period between the procedure and the follow-up visit.

### 2.3. Catheter Ablation Technique

The catheter ablation procedure consisted of endocardial ostial isolation of the antrum of each pulmonary vein using radiofrequency. A Lasso™ catheter (Biosense Webster, Irvine, CA, USA) and a Celsius™ Thermo Cool irrigated tip (Biosense Webster, Irvine, CA, USA) were used, according to current recommendations [14]. 

Transesophageal echocardiography and computed tomographic scan were performed prior to the ablation procedure in all patients in order to rule out left atrial thrombi and to obtain pulmonary vein morphology for the electroanatomic mapping system. Intravenous heparin 100 IU/kg was administered after a successful transseptal puncture, followed by a constant dose of 1000 IU/h, irrespective of oral anticoagulation regimen. During the procedure, patients underwent conscious sedation. Pulmonary vein isolation was the standard approach in all patients. Radiofrequency ablation was performed using a three-dimensional electroanatomic mapping system (CARTO; Biosense Webster, CA or NavX/Velocity; Abbott, St. Paul, MN, USA). After double transseptal punctures, the irrigated radiofrequency ablation catheter and a circular mapping catheter were positioned into the left atrium. Radiofrequency energy was delivered at a distance of 5–10 mm from the pulmonary vein ostia (at the pulmonary vein antrum) using 35 W radiofrequency energy.

In patients with paroxysmal atrial fibrillation longer than 24 h, the ablation procedure was completed by complex fractionated atrial electrogram and cavo-tricuspid isthmus ablation (if common atrial flutter was confirmed before or during the procedure) according to the decision of the electrophysiologist. The procedure was performed under local anesthesia by femoral access and deep sedation with fractionated intravenous morphine during radiofrequency application. During the ablation procedure, esophageal temperature monitoring was not available.

### 2.4. Upper Gastrointestinal Endoscopy

Gastroenterological assessment by upper gastrointestinal endoscopy was performed in the 48–72 h before the atrial fibrillation ablation procedure using the Olympus Exera CV-160 endoscope (Global Endoscopy Solutions, Inc. Corporate Headquarters; 951 North Volusia Ave STE 200 Orange City, FL 32763). Afterwards, the examination was repeated 3 months after the procedure. All the endoscopic evaluations were performed by one single senior gastroenterologist, with significant experience in upper gastrointestinal endoscopy. The presence and grade of esophagitis (described according to the Los Angeles classification [16]: grade A esophagitis is defined as at least one break in the esophageal mucosa, no longer than 5 mm and which does not extend between two folds; grade B esophagitis is defined by a break that is over 5 mm long, but does not extend between the tops of two folds; grade C esophagitis is described in the presence of at least one mucosal break which is continuous between two or more mucosal folds, but affecting less than 75% of the esophageal circumference; grade D esophagitis is present when the changes involve at least 75% of the circumference) and Barrett’s esophagus were noted. The gastroenterologist screened for newly developed typical symptoms suggestive of gastro-esophageal reflux (heartburn and/or regurgitation) or the need for acid-suppressive medication (such as proton pomp inhibitors) during the follow-up period. They counselled patients to report any dyspeptic symptoms between scheduled visits. 

### 2.5. Bidimensional Transthoracic Echocardiography

Transthoracic echocardiographic measurements were done using a Sonoscape SSI 8000 Ultrasound Machine (Providian Medical Equipment LLC, Chagrin Falls, OH, USA). The parameters assessed during echocardiographic examination were the left ventricular systolic function by left ventricular ejection fraction (by the Simpson method) and the diastolic left ventricular function parameters (E/A, E/e’, left atrium area). E/A ratio was assessed in patients in sinus rhythm, in apical 4- or 2-chamber (E represents the maximum velocity of early diastolic filling of the mitral flow and A stands for the maximum velocity of late diastolic filling of mitral flow). The diastolic index or E/e’ ratio (the ratio of early mitral inflow to tissue velocity of the mitral annulus) was assessed in all patients in apical 4- or 2-chamber. We used the e’ velocity obtained by tissue Doppler imaging from the septal and lateral mitral annulus. In patients with atrial fibrillation, during the echocardiographic examination, we used average velocity measurements from 10 consecutive cycles.

Left ventricular systolic and diastolic function (by left ventricular ejection fraction, E/A ratio and E/Em ratio) and left atrium area (as a marker of structural remodeling of the left atrium) were assessed according to the current recommendations [17]. All measurements were conducted by one single experienced operator (with transthoracic echocardiography European Association of Cardiovascular Imaging—EACVI accreditation).

### 2.6. Ethics

This study was approved by the Hospital and University Ethics Committee and complies with the Declaration of Helsinki. All patients provided written informed consent for participation in the study.

### 2.7. Statistical Analysis

Categorical data are presented as frequencies and percentages; continuous variables are expressed as mean ± standard deviation. Categorical, ordinal and numerical variables were compared between groups using χ^2^, Cochran and Wilcoxon rank sum tests, Kruskal–Wallis and Anova test, respectively. All statistical tests were two-tailed and performed with SPSS 15.0 (SPSS Inc., Chicago, IL, USA). A *p*-value < 0.05 was considered statistically significant.

## 3. Results

Seventy-five eligible participants completed the study and were included in the final analysis: 46 subjects in the study group (patients who underwent atrial fibrillation catheter ablation) and 29 patients in the control group (who received standard medical treatment, without undergoing atrial fibrillation ablation). The baseline characteristics of participants in both groups are presented comparatively in Table 1.

The subjects referred for atrial fibrillation ablation were significantly younger (*p* = 0.001), predominantly male (*p* = 0.03) and with higher body mass index (*p* = 0.046) compared to controls. When comparing pro-BNP levels between study patients and controls, the values were significantly higher in the control group (344 ± 180 vs. 760 ± 253 pg/mL, respectively; *p* = 0.001), although no differences were noted in terms of left atrium size. The frequency of typical symptoms suggestive of gastro-esophageal reflux and esophagitis did not differ significantly at baseline between the two groups. 

### 3.1. Study Subjects vs. Controls Three Months after Atrial Fibrillation Ablation

The percentage of patients with sinus rhythm in the study group compared to controls (under antiarrhythmic medication) was 88.9% vs. 57.1%, respectively (*p* = 0.009). Comparative data on echocardiographic and gastroenterological parameters at three months after atrial fibrillation ablation are presented in detail in Table 2.

In the study group, one additional participant developed typical symptoms suggestive of gastro-esophageal reflux, as opposed to the control group, where six additional subjects presented with typical symptoms of reflux three months after the initial evaluation.

The follow-up echocardiographic evaluation revealed a significantly lower left ventricular ejection fraction in subjects who did not undergo atrial fibrillation ablation (*p* = 0.048). The left ventricular diastolic function was also modified in the controls, as revealed by the E/A ratio.

The upper gastrointestinal endoscopic evaluation found esophagitis to be significantly more frequent in participants who underwent the atrial fibrillation catheter ablation procedure (*p* = 0.001). The severity of the esophagitis in all affected patients was grade A, defined according to the Los Angeles classification as one or more mucosal breaks confined to the mucosal folds, each less than 5 mm in maximum length [16]. None of the study participants presented with Barrett’s esophagus.

### 3.2. Patients with Symptomatic Gastro-Esophageal Reflux

In a subgroup analysis of participants with typical symptoms suggestive of gastro-esophageal reflux, no significant differences were noted in terms of sinus rhythm prevalence at three months (89.5% in the atrial fibrillation ablation group vs. 88.5% in the control group; *p* = 0.709). Comparative data in patients with symptomatic gastro-esophageal reflux is presented in Table 3.

Left ventricular ejection fraction was still significantly lower in control subjects compared to those who underwent atrial fibrillation ablation (*p* = 0.016). In addition to that, left ventricular diastolic function (evaluated by the E/A ratio) was also significantly different between the two groups (*p* = 0.036).

Upper digestive endoscopy at three months found that esophagitis remained significantly more frequent in the group of patients who underwent the atrial fibrillation ablation procedure (*p* = 0.049). 

## 4. Discussion

Both gastro-esophageal reflux and atrial fibrillation are frequently encountered diseases in the general population, with significant impacts on the quality of life of patients and burdensome on healthcare services. Since the endorsement of radiofrequency catheter ablation for atrial fibrillation as a curative technique, the cardiologist’s point of view about the relationship between this cardiac arrythmia and gastro-esophageal reflux disease has been more elaborately expressed in the literature [18,19,20,21,22]. 

**Figure 2:**
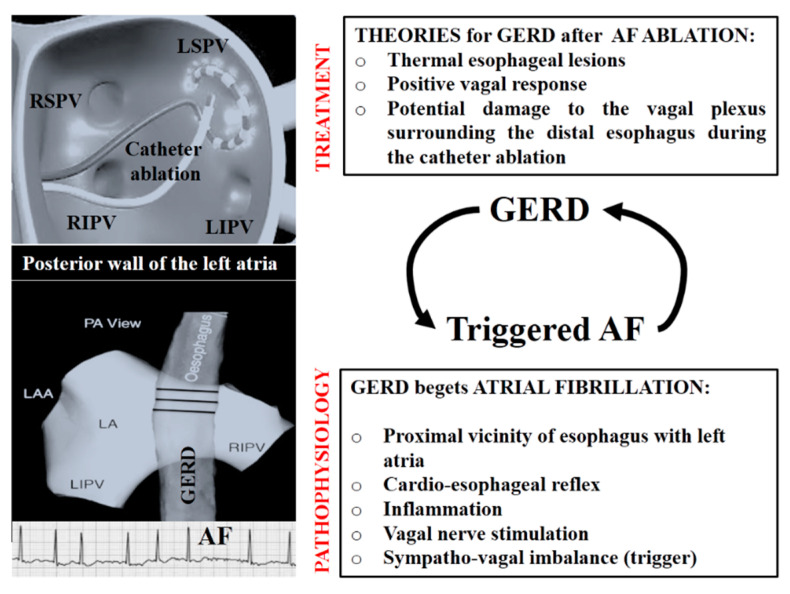
The complex relationship between GERD and AF from a pathophysiological and therapeutic point of view. GERD = gastroesophageal reflux disease; LAA = left atrial appendage; LA = left atrium; vein; LSPV= left superior pulmonary vein; LIPV= left inferior pulmonary vein; PA = posterior-anterior; RIPV= right inferior pulmonary vein; RSPV = right superior pulmonary vein.

The relationship between these two clinical entities is quite complex and recent studies suggest it might be bidirectional. A summary of the main pathophysiological considerations highlighted in the literature is schematically presented in Figure 2.

Firstly, the presence of gastro-esophageal reflux in patients with atrial fibrillation has been studied as a possible trigger for this arrhythmia [19,21]. In addition to the mechanical effect due to the close positioning of the esophagus and left atrium, the relationship between atrial fibrillation and gastro-esophageal reflux may also imply additional pathophysiological mechanisms. Some of the proposed explanations are centered around chronic inflammation, local esophageal mucosal inflammation (that may affect the adjacent vagal nerves and local afferent–efferent reflexes) and autonomic imbalances that may initiate and perpetuate atrial fibrillation or the releasing of inflammatory mediators [7]. Therefore, this complex relationship, which seems to be bidirectional, is far from being understood.

On the other hand, catheter ablation implying the use of radiofrequency in the vicinity of the esophagus might increase the risk of esophageal injury. This interventional technique used for the treatment of atrial fibrillation may cause esophageal lesions and vagal nerve damage by thermal injury in up to 47% of cases [22,23]. Esophageal ulcerations, potential precursors of dramatic life-threatening complications such as atrio-esophageal fistula or motility disorders such as “jackhammer” esophagus have also been described as possible consequences of radiofrequency catheter ablation performed in the left atrium [20]. Additional potential mechanisms for esophageal lesions include chronic esophageal mucosal inflammation that may affect the adjacent vagal nerves, local afferent–efferent reflex mechanisms and release of inflammatory mediators. From a therapeutic standpoint, research has found that post-procedural esophageal lesions may be completely resolved with acid-suppressive medication at 2–4 weeks following the ablation [24]. 

Injuries to the vagal nerve have also been documented as potential side effects of AF ablation. Symptoms such as nausea, vomiting or abdominal pain that generally occur during the first several hours following the procedure are frequently linked to this [25,26]. A recent study by Grosse Meininghaus et al. found that there may be a bidirectional relationship between vagal nerve injury and (peri-)esophageal lesions [27]. Research suggests that food retention, periesophageal edema and mucosal ulcers may be more common in subjects who experienced vagal damage; at the same time, pre-existing esophagitis is associated with a greater likelihood of vagal nerve injury [27]. 

In this small prospective study, we did not find typical symptoms suggestive of gastro-esophageal reflux to be more frequent at three months in patients who underwent catheter ablation for atrial fibrillation compared to controls. On the other hand, endoscopically proven esophagitis (not more severe than grade A Los Angeles) was significantly more common in these patients. One explanation for this finding is that esophagitis may be present even in patients with asymptomatic gastro-esophageal reflux [28,29]. However, it is important to note that currently only grade C or D Los Angeles esophagitis are considered definitive endoscopic criteria for gastro-esophageal reflux according to the Lyon Consensus [30]. These results highlight the need for further research, which focuses on asymptomatic gastro-esophageal reflux and esophagitis in patients undergoing ablation for paroxysmal atrial fibrillation. 

Noteworthy, it is important to mention the fact that in this study (grant no. POSDRU/159/1.5/S/133377—CHRONEX), the patient enrollment took place before the 2017 guidelines (which recommend that acid-suppressive medication should be administered for 2–3 months after the ablation procedure) were published [31].

Another controversial topic in the recent literature addresses the potential increase in esophageal acid exposure after pulmonary vein antrum isolation. A study by Martinek et al. found that a significant number of patients who underwent radiofrequency catheter ablation developed pathologic acid reflux at 24 h after the procedure [8]. However, this hypothesis was not confirmed in the setting of another prospective study carried out on 25 subjects at baseline and after atrial fibrillation ablation [32]. Therefore, this issue remains controversial, emphasizing the need for assessment by impedance-pH monitoring in future studies.

The early recognition and active management of these lesions are essential steps which may improve the outcomes of these patients. Esophageal temperature monitoring has been proposed as a potentially useful tool; however, its role in reducing the incidence of esophageal injury is still unclear [33]. Moreover, additional factors such as patient characteristics and various radiofrequency energy delivery strategies should also be carefully considered [34]. In this regard, the pre-procedural assessment of the position of the esophagus using computer tomography or magnetic resonance imaging is recommended [35]. Moreover, reduced energy application on the posterior wall of the left atrium may be a superior approach, given its anatomical proximity to the esophagus [35]. 

In this prospective study, the presence of symptomatic gastro-esophageal reflux did not seem to affect the short-term post-procedural outcome in the atrial fibrillation ablation group. In the group who underwent catheter ablation, there were no significant differences in terms of sinus rhythm prevalence in patients with symptomatic gastro-esophageal reflux compared to those without.

There are some differences in the baseline characteristics of patients included in this study, which may influence the results. In the study group, the percentage of male participants and mean BMI were significantly higher. Data from the literature suggest that the prevalence of atrial fibrillation is higher in men compared to women in all age groups [36]. In addition to that, the referral of women for atrial fibrillation ablation tends to be less frequent and delayed compared to men [37,38]. This may be due to the fact that male patients with atrial fibrillation are usually more symptomatic; therefore, they are more interested in undergoing paroxysmal atrial fibrillation ablation, which is considered a curative procedure for this arrhythmia. The mean age is another baseline characteristic that differed significantly between the two groups. The current guidelines recommend that the ablation procedure is performed for younger, otherwise healthy patients with symptomatic paroxysmal atrial fibrillation refractory to one or more antiarrhythmic medications [13]. This may be the likely explanation for the significantly lower mean age in subjects included in the study group compared to controls. 

These findings are especially important as some of the parameters that varied significantly between the two groups may also play a role in GERD pathophysiology. For example, a recent systematic review has found that obesity is associated with a higher prevalence of GERD, and weight loss may be beneficial in this regard. Additionally, age also seems to have an impact on the GERD prevalence, symptomatology and severity of esophageal lesions [39,40]. In this context, these results should be interpreted with caution.

The importance of this study lies in the fact that it provides additional data on the relationship between gastro-esophageal reflux disease and atrial fibrillation, which is still very controversial and poorly understood. This small prospective study sought to investigate the presence of typical symptoms suggestive for gastro-esophageal reflux and esophagitis early following atrial fibrillation ablation in naïve patients (without any prior gastroenterological evaluation or treatment). Whether the early diagnosis of gastro-esophageal reflux or esophagitis may lead to better outcomes after atrial fibrillation ablation is still a highly debated topic. Some authors suggest that a short-term treatment with proton pomp inhibitors in the first 3 months following the ablation procedure might prevent atrial fibrillation recurrences and esophagitis-related complications [41,42]. 

### Study Limitations

This study has several limitations. First and foremost, it is important to note that the number of included participants was relatively small, thus the results of the statistical analysis could have been influenced by the study size. We acknowledge this as one of the main limitations of our study and highlight the need for further research to be conducted with appropriate sample sizes. These results should be interpreted with caution and should be confirmed by larger trials. In addition to that, as previously mentioned, the two groups were quite different in terms of baseline characteristics, such as age, gender and body mass index. One possible explanation that may account for some of these differences resides in the fact that the guidelines recommend the paroxysmal atrial fibrillation ablation procedure in younger subjects, whilst also taking into account the personal preference of the patients for this method. Another important limitation of the current study is the fact that participants were not evaluated by impedance-pH monitoring or high-resolution manometry in order to rule out asymptomatic and non-erosive gastro-esophageal reflux or esophageal motility disorders. The main focus of this study was solely the presence of typical symptoms suggestive for gastro-esophageal reflux. Therefore, expanding the clinical research in this field for a better understanding of the relationship between atrial fibrillation and gastro-esophageal reflux is essential, keeping in mind the aforementioned directions for future studies.

## 5. Conclusions

In this prospective study, at three months following the procedure, typical symptoms suggestive of gastro-esophageal reflux were not more frequently encountered in patients who underwent catheter ablation for paroxysmal atrial fibrillation. On the other hand, in this pilot study, esophagitis (no more severe than grade A Los Angeles) tended to be more common in the study group compared to controls at three months post-procedure. In the group who underwent catheter ablation, there were no significant differences in terms of sinus rhythm prevalence in patients with symptomatic gastro-esophageal reflux compared to those without. Expanding the research in this field in order to gain a better understanding of the relationship between atrial fibrillation and gastroesophageal reflux disease is highly encouraged.

## Figures and Tables

**Table 1:** Baseline patient characteristics.

Parameter	Study Group N = 46	Control Group	
		N = 29	*p* Value
Age (years)	57.76 ± 7.66	67.81 ± 8.52	**0.001**
Male (%)	28 (62.2)	7 (33.3)	**0.030**
BMI (kg/m^2^)	28.96 ± 3.12	26.81 ± 5.19	**0.046**
Smoking (%)	18 (39.1)	10 (34.4)	0.07
Dyslipidemia (%)	31 (68.8)	14 (66.6)	0.918
Hypertension (%)	29 (64.4)	19 (90.5)	**0.028**
Diabetes mellitus (%)	8 (17.7)	4 (19.0)	0.827
Symptomatic GERD (%)	19 (42.2)	13 (61.9)	0.220
Esophagitis (%)	12 (26.7)	8 (38.1)	0.513
Hiatal hernia (%)	12 (26.7)	5 (23.8)	0.956
Left atrium area (cm^2^)	26.51 ± 5.31	26.47 ± 5.33	0.766
Pro-BNP (pg/mL)	344 ± 180	760 ± 253	**0.001**

Data are frequency counts (percentage of total) or mean ± standard deviation. BMI = body mass index; BNP = brain natriuretic peptide; GERD = gastroesophageal reflux disease.

**Table 2:** Echocardiography parameters at three months after atrial fibrillation catheter ablation.

Parameter	Study Group		Control Group		*p* Value *
	With GERD N = 20	Without GERD N = 26	With GERD N = 19	Without GERD N = 10	
**ECHOCARDIOGRAPHY**					
E/A ratio	1.27 ± 0.43	1.53 ± 0.68	0.89 ± 0.48	1.89 ± 0.49	**0.046**
E/Em ratio	7.90 ± 1.47	8.41 ± 3.43	9.61 ± 2.77	8.88 ± 5.14	0.475
E wave deceleration time (ms)	219.17 ± 41.04	203.75 ± 41.13	239.58 ± 62.28	192.86 ± 54.53	0.222
Left atrium area (cm^2^)	25.34 ± 5.06	27.26 ± 5.21	26.31 ± 5.13	27.30 ± 5.06	0.625
Left ventricle ejection fraction (%)	59.52 ± 6.87	58.36 ± 7.79	53.06 ± 8.17	54.50 ± 7.62	**0.048**

* Kruskal–Wallis Test. E/A ratio = E wave velocity/A wave velocity ratio; E/Em ratio = E wave velocity/Em velocity ratio; GERD = gastroesophageal reflux disease.

**Table 3:** Echocardiographic and gastroenterological parameters in patients with symptomatic GERD at 3 months after atrial fibrillation ablation.

Parameter	Symptomatic GERD		*p* Value *
	With AF Ablation (N = 20)	Without AF Ablation (N = 19)	
**ECHOCARDIOGRAPHY**			
E/A ratio	1.27 ± 0.43	0.89 ± 0.48	**0.036**
E/Em ratio	7.90 ± 1.47	9.61 ± 2.77	**0.034**
E wave deceleration time (ms)	219.17 ± 41.04	239.58 ± 62.28	0.480
Left atrium area (cm^2^)	25.34 ± 5.06	26.31 ± 5.33	0.585
Left ventricle ejection fraction (%)	59.52 ± 6.87	53.06 ± 8.17	**0.016**
**UPPER GASTROINTESTINAL ENDOSCOPY**			
Esophagitis (grade A LA) (%)	55.0	43.8	0.049 *

* Kruskal–Wallis Test. Data are frequency counts (percentage of total) or mean ± standard deviation. AF = atrial fibrillation; E/A ratio = E wave velocity/A wave velocity ratio; E/Em ratio = E wave velocity/Em velocity ratio; GERD = gastroesophageal reflux disease; LA = Los Angeles classification.
